# Exploiting docetaxel-induced tumor cell necrosis with tumor targeted delivery of IL-12

**DOI:** 10.1007/s00262-023-03459-7

**Published:** 2023-05-11

**Authors:** S. Elizabeth Franks, Ginette S. Santiago-Sanchez, Kellsye P. Fabian, Kristen Solocinski, Paul L. Chariou, Duane H. Hamilton, Joshua T. Kowalczyk, Michelle R. Padget, Sofia R. Gameiro, Jeffrey Schlom, James W. Hodge

**Affiliations:** Center for Immuno-Oncology, Center for Cancer Research, National Cancer Institute, National Institutes of Health, Bldg. 10, Rm 8B13, 10 Center Drive, Bethesda, MD 20892 USA

**Keywords:** NHS-IL-12, Docetaxel, rIL-12, Necrosis, Immunotherapy, Combination immunotherapy

## Abstract

There is strong evidence that chemotherapy can induce tumor necrosis which can be exploited for the targeted delivery of immuno-oncology agents into the tumor microenvironment (TME). We hypothesized that docetaxel, a chemotherapeutic agent that induces necrosis, in combination with the bifunctional molecule NHS-IL-12 (M9241), which delivers recombinant IL-12 through specific targeting of necrotic regions in the tumor, would provide a significant antitumor benefit in the poorly inflamed murine tumor model, EMT6 (breast), and in the moderately immune-infiltrated tumor model, MC38 (colorectal). Docetaxel, as monotherapy or in combination with NHS-IL-12, promoted tumor necrosis, leading to the improved accumulation and retention of NHS-IL-12 in the TME. Significant antitumor activity and prolonged survival were observed in cohorts receiving docetaxel and NHS-IL-12 combination therapy in both the MC38 and EMT6 murine models. The therapeutic effects were associated with increased tumor infiltrating lymphocytes and were dependent on CD8^+^ T cells. Transcriptomics of the TME of mice receiving the combination therapy revealed the upregulation of genes involving crosstalk between innate and adaptive immunity factors, as well as the downregulation of signatures of myeloid cells. In addition, docetaxel and NHS-IL-12 combination therapy effectively controlled tumor growth of PD-L1 wild-type and PD-L1 knockout MC38 in vivo, implying this combination could be applied in immune checkpoint refractory tumors, and/or tumors regardless of PD-L1 status. The data presented herein provide the rationale for the design of clinical studies employing this combination or similar combinations of agents.

**Supplementary Information:**

The online version contains supplementary material available at 10.1007/s00262-023-03459-7.

## Introduction

In 2018, nearly 80% of patients diagnosed with advanced or metastatic cancer were eligible to receive conventional chemotherapy [1]. In recent years, evidence has shown select chemotherapy regimens induce immunogenic cell stress, ranging from immunogenic modulation to immunogenic cell death [2], as well as necrosis, which is immunogenic in nature through plasma membrane rupture and release of damage associated molecular patterns [3–5]. Included in these select chemotherapy regimens that cause immunogenic cell stress is docetaxel [6, 7], a taxane that functions to stabilize microtubules, leading to mitotic catastrophe, resulting in apoptotic or necrotic cell death [8–10]. Docetaxel is among the most widely used conventional chemotherapies [11], with FDA approval in breast cancer, non-small cell lung cancer, castration-resistant prostate cancer, gastric adenocarcinoma, and squamous cell carcinoma of the head and neck.

Interleukin-12 (IL-12), a pleiotropic proinflammatory cytokine, is produced by antigen presenting cells and plays a critical role in the crosstalk between innate and adaptive immunity [12]. IL-12 promotes cell-mediated immunity by inducing CD4^+^ Th1 cell differentiation and IFNγ production and enhancing recruitment and cytolytic effector function of both CD8^+^ T cells and natural killer (NK) cells [13, 14]. Given these pleiotropic effects that promote multifaceted antitumor immunity, IL-12 was an attractive target in preclinical investigation. However, systemic administration of recombinant human IL-12 (rhIL-12) ultimately failed to achieve such promise in clinical trials due to severe dose limiting toxicities [13–15].

More recently, interest in IL-12 has reemerged under a new paradigm where IL-12 is targeted to the tumor microenvironment (TME), thereby enhancing local antitumor immunity while ameliorating toxicities and risks inherent to systemic administration [15]. NHS-IL-12 (M9241) is a recombinant fusion protein that achieves targeted delivery of this cytokine to areas of tumor necrosis through fusion of two molecules of IL-12 to the C-terminus of the NHS76 antibody, which possesses specificity to ssDNA and dsDNA [12]. In comparison to recombinant murine IL-12 (rmIL-12), preclinical studies of NHS-IL-12 have shown enhanced safety and efficacy, and synergistic effects when used in combination with conventional and targeted chemotherapies [16–22]. These studies have paved the way for ongoing clinical trials where NHS-IL-12 monotherapy was well tolerated in a Phase I clinical trial [23], with additional trials currently evaluating NHS-IL-12 in combination with several immuno-oncology (IO) agents [24, 25].

Herein, we present for the first time that the synergy observed with NHS-IL-12 and docetaxel in combination is due to docetaxel-induced tumor necrosis. Because the antigen recognition domain of NHS76 is directed against areas of necrosis, and is therefore tumor targeted, we observed enhanced NHS-IL-12 retention in the TME, resulting in improved antitumor immunity, and increased infiltration of CD4^+^ and CD8^+^ T cells.

## Materials and methods

### Cell lines

Murine breast (EMT6) carcinoma cells were obtained from American Type Culture Collection (ATCC, Manassas, Virginia, USA) and were cultured in the recommended media. Murine colon (MC38) carcinoma cells were cultured as described [26]. All cell lines were free of *Mycoplasma,* confirmed via MycoAlert Mycoplasma Detection Kit (Lonza, Basel, Switzerland), cultured at 37 °C with 5% CO_2,_ and were used at low passage numbers. MC38-PD-L1 knockout (KO) tumor cells were generated in our laboratory by co-transfection of MC38 cells with a recombinant Cas9 protein version 2 and a TrueGuide Synthetic guide RNA targeting murine PD-L1 (ID number CRISPR228292_SGM) using the Lipofectamine CRISPRMAX transfection reagent (ThermoFisher Scientific, Waltham, MA, USA) following the manufacturer’s recommended protocol. MC38-PD-L1 negative cells were cloned and used in subsequent studies.

### Animals and tumor models

C57BL/6 and Balb/c mice were bred and maintained at the National Institutes of Health (NIH) (Bethesda, MD, USA) and were housed in microisolator cages under specific pathogen-free conditions. All animal procedures reported in this study that were performed by National Cancer Institute (NCI)–Center for Cancer Research affiliated staff were approved by the NCI Animal Care and Use Committee (ACUC) and in accordance with federal regulatory requirements and standards utilizing ARRIVE1 reporting guidelines [27]. NHS-IL-12 (M9241) was obtained from EMD Serono (Rockland, MA, USA) through a Cooperative Research and Development Agreement (CRADA) with the NCI, NIH (Bethesda, MD, USA).

For docetaxel and NHS-IL-12 combination strategies, 8–16 week-old female mice were inoculated with MC38 [3 × 10^5^ cells/mouse subcutaneously (s.c.)], MC38-PD-L1 KO (3 × 10^5^ cells/mouse s.c) in the right flank of C57Bl/6 mice or EMT6 tumor cells (3 × 10^5^ cells/mouse s.c) in the right flank of Balb/c recipients. Treatment initiation occurred on day 7, or when mean tumor volume was between 50 and 100 mm^3^. Where indicated, MC38, MC38-PD-L1) KO and EMT6 tumor-bearing mice were treated with 500 µg docetaxel, delivered intraperitoneally (i.p.) on days 7, 9 and 11. NHS-IL-12, unless otherwise noted, was administered at 2 µg s.c. between the shoulder blades on days 10, 12 and 14.

For comparative studies performed with rIL-12, 8–16 week-old female mice were inoculated with MC38 tumor cells (3 × 10^5^ cells/mouse s.c.) in the right flank of C57Bl/6 mice. MC38 tumor-bearing mice were treated with docetaxel (500 µg, i.p) on days 7, 9 and 11 and NHS-IL-12 (2 µg, s.c.) or rIL-12 (1 µg, s.c.) (R&D System, Minneapolis, MN, USA) on days 10, 12 and 14.

For depletion experiments, 8–16 week-old female mice were inoculated with MC38 tumor cells (3 × 10^5^ cells/mouse s.c.) in the right flank of C57Bl/6 mice. Anti-CD4 [GK 1.5, 100 µg, (i.p.)]; anti-CD8 [2.43, 100 µg, (i.p.); BioXcell, Lebanon, NH, USA)] and anti-NK1.1 [100 µg, (i.p.); (PK136, BioXCell)] were injected on days 3, 4, 5, 12, and 19 post-tumor inoculation. Treatment initiation occurred on day 7, or when mean tumor volume was between 50 and 100 mm^3^. MC38 tumor-bearing mice were treated with docetaxel (500 µg, i.p) on days 7, 9 and 11 and NHS-IL-12 (2 µg, s.c.) on days 10, 12 and 14.

### Necrosis and NHS-IL-12 quantification

MC38 tumors were harvested 17 days post tumor inoculation, preserved in Z-fix (Anatech, Richmond, British Columbia, Canada) and paraffin embedded. Tumor sections were H&E stained per standard procedures (VitroVivo Biotech, Gaithersburg, MD, USA). Tumor sections were imaged using an AxioScan Z1 Slide Scanner (Zeiss, Oberkochen, Germany). Percentages of necrosis were quantified by a blinded board-certified pathologist [28, 29].

For quantification of NHS-IL-12 in the tumor and in the periphery, the human Fc portion of NHS was detected via Human IgG ELISA (Sigma-Aldrich, St. Louis, MO, USA), per manufacturer protocol. Generation of tumor supernatant was achieved through homogenization of tumors in PBS using the gentleMACS Dissociator (Miltenyi Biotec, Bergisch Galdbach, Germany). Supernatants and serum were stored at -20ºC until analysis.

### Peripheral cytokine analyses

Serum collection was performed at indicated time points (refer to Figs. 2A and 3A). Serum IFNγ, IL-12, TNFα, and KC/GRO were quantified using the murine V-Plex Proinflammatory Panel 1 kit and MESO QuickPlex SQ 120 (Meso Scale Diagnostics; Rockville, Maryland, USA), according to the manufacturer’s instructions.

### OPAL immunofluorescence

Tumor tissue was fixed in Z-fix (Anatech) and paraffin embedded. Slides with tumors from treated and untreated MC38 mice were stained using Opal 4-Color Manual IHC Kit (Akoya Biosciences; Marlborough, MA, USA) according to the manufacturer’s instructions. Antibodies used included anti-CD8 (ab217344, Abcam, Waltham, MA, USA), anti-CD4 (ab183685, Abcam), and anti-FoxP3 (ab75763, Abcam). Slides were scanned with Axio Scan.Z1 and Zen software (Zeiss). Ten representative images from each tumor of each group were collected for analysis. Stained tumors were analyzed using automated cell counting based on Otsu thresholding and quantification of number of nuclei in each region of interest. Regulatory T cells [30] were determined by dual expression of CD4 and Foxp3. Secondary-only staining was used as a negative control.

### RNA sequencing and analysis

Total tumor RNA was isolated at day 17 post MC38 tumor inoculation using the RNeasy Mini kit (Qiagen; Germantown, MD, USA). Tumor bulk RNA was sequenced by Novogene UC Davis Sequencing Center (Novogene Corporation, Ltd, Davis, CA, USA).

Bioinformatics analysis and visualization were performed with the NIH Integrated Analysis Portal (NIDAP) using R programs developed on the Foundry platform (Palantir Technologies, Inc, Denver, CO, USA).

### Statistical analyses

Student *t* test was used to compare two groups. One-way or two-way ANOVA was performed to compare more than two groups with Tukey’s post hoc analysis for correction. Outliers were identified by Grubb’s test. Log-rank (Mantel-Cox) test was used to determine survival proportions. Fisher’s test for pathways applied top 10% genes with p value cut-off of *p* = 0.05. *P* values less than 0.05 were considered significant with *p < 0.05, **p < 0.01, ***p < 0.001, ****p < 0.0001. Error bars in figures represent mean ± SEM. GraphPad Prism 9.0 (San Diego, CA, USA) was utilized for analyses.

## Results

### Treatment with docetaxel induces necrosis in MC38 tumors

**Fig. 1:**
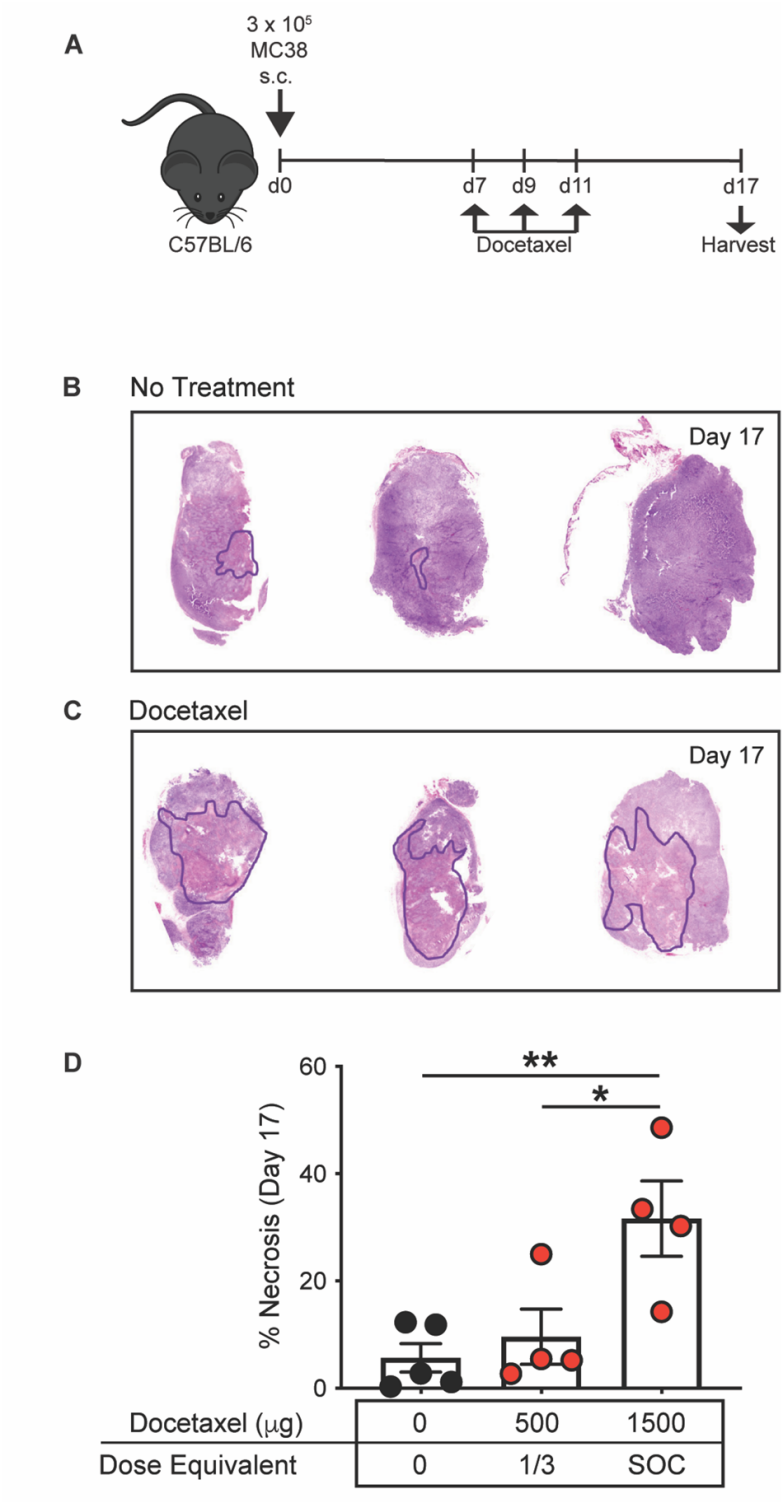
Treatment with docetaxel induces necrosis in MC38 tumors. (**A**) Graphical representation of experimental design. Representative H&E stains showing necrotic areas (outlined in black) in MC38 tumors harvested from **(B)** untreated animals or (**C**) animals treated with docetaxel on day 17 post-tumor inoculation. (**D**) Percentage necrosis was quantified by a blinded board-certified pathologist. Quantification of percent necrosis in tumors from animals treated with 500 µg (*n* = 4) and 1500 µg (*n* = 4) docetaxel and untreated controls (*n* = 5). SOC = standard of care. *p < 0.05, **p < 0.01

To determine if docetaxel induces necrosis within the tumor, we treated MC38 tumor-bearing mice, a “warm” colorectal cancer tumor model [31], with docetaxel and analyzed tumors for necrosis 6 days after the last treatment (Fig. 1A). The tumors were excised, H&E stained, and areas of necrosis were identified (outlined in black). Although MC38 tumors from untreated animals had a baseline level of necrosis (Fig. 1B), animals that received docetaxel had a significant increase in tumor necrosis compared to the untreated group (*p* = 0.009; Fig. 1C, D).

Docetaxel induced necrosis in a dose-dependent manner, with animals that received only one dose of docetaxel [500 μg = 1/3 standard-of-care dose (SOC)] having a 1.7-fold increase in areas of necrosis in comparison to untreated controls (Fig. 1D). Animals that received three doses of docetaxel (1500 μg = SOC; 75 to 100 mg/m^2^) had a 5.6-fold increase in necrosis in comparison to untreated controls (*p* = 0.009; Fig. 1D). Mice given the full SOC treatment regimen showed a 3.4-fold increase in necrosis in comparison to animals that received one dose of docetaxel (*p* = 0.03; Fig. 1D). These data, taken together, show that docetaxel induced necrosis in a dose-dependent manner.

### Docetaxel-induced necrosis increases NHS-IL-12 retention in the tumor microenvironment

**Fig. 2:**
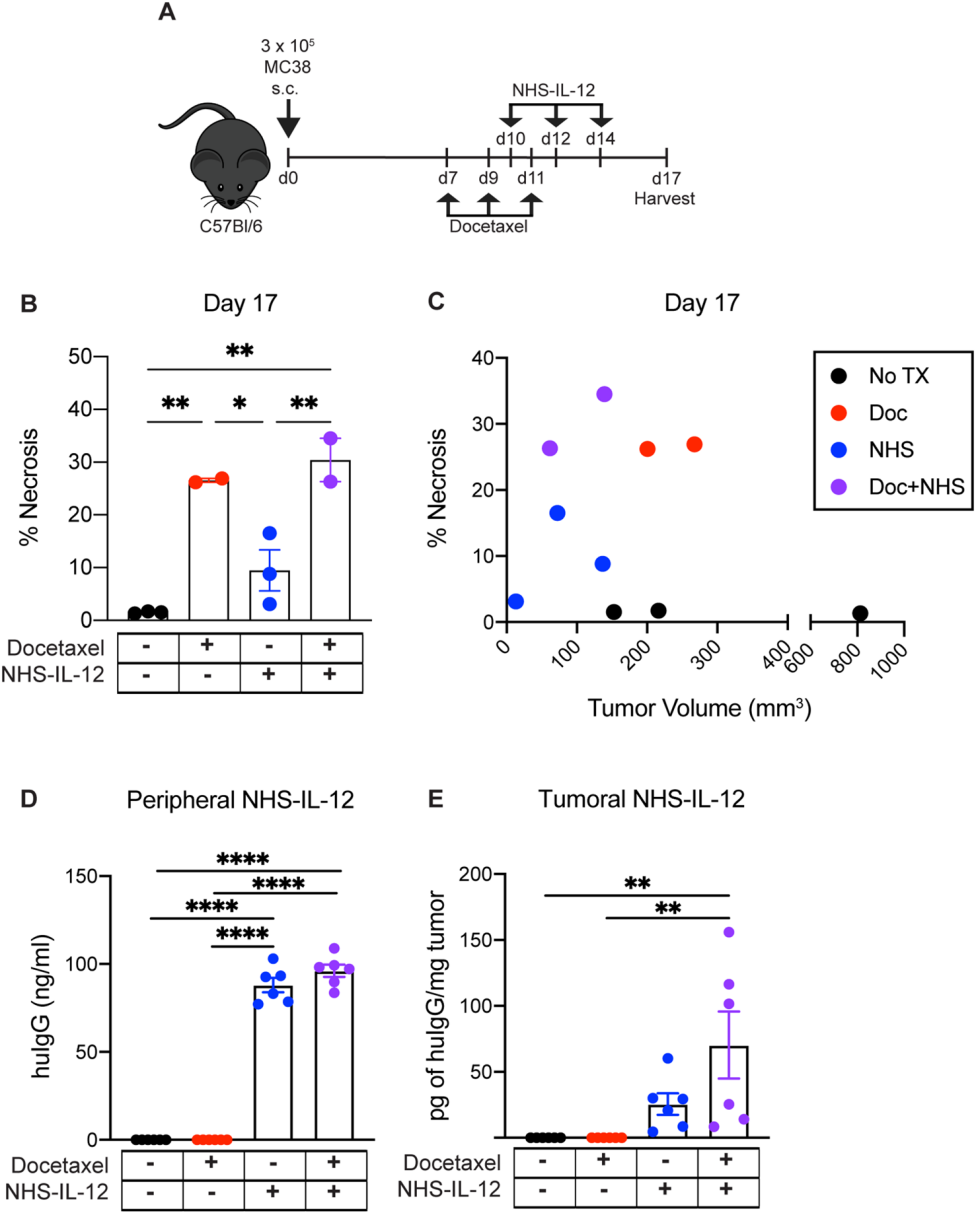
Docetaxel-induced necrosis increases NHS-IL-12 retention in the tumor microenvironment. (**A**) Graphical representation of experimental design. (**B**) Quantification of percent necrosis of MC38 tumor-bearing animals that were untreated (black symbols; *n* = 3) or received docetaxel monotherapy (red symbols; *n* = 2), NHS-IL-12 monotherapy (blue symbols; *n* = 3) or docetaxel + NHS-IL-12 combination therapy (purple symbols; *n* = 2). (**C**) Evaluation of percent necrosis as a function of tumor volume of untreated, docetaxel, NHS-IL-12, and docetaxel + NHS-IL-12 combination therapy. Enumeration of NHS-IL-12 via ELISA found in the (**D**) periphery and (**E**) intratumorally of untreated animals (*n* = 5) and animals receiving docetaxel (*n* = 5), NHS-IL-12 (*n* = 6), and docetaxel + NHS-IL-12 combination therapy (*n* = 6). Doc = docetaxel. huIgG = human IgG. *p < 0.05, **p < 0.01, ***p < 0.001, ****p < 0.0001

NHS-IL-12 is a bifunctional molecule with two murine IL-12 molecules fused to the Fc region of the human IgG1 monoclonal antibody, NHS76 [12]. The antigen recognition domain of NHS76 is specific for DNA/histones, which are exposed in necrotic areas of tumors [12]. To determine if docetaxel-induced necrosis allows for increased retention of NHS-IL-12 in the TME, we first evaluated levels of tumor necrosis of MC38 tumor-bearing mice treated with docetaxel and NHS-IL-12 as monotherapies and in combination (Fig. 2A). Percentages of necrosis were quantified by a blinded board-certified pathologist. Animals that received docetaxel monotherapy (red symbols) and docetaxel + NHS-IL-12 combination therapy (purple symbols) showed a significant increase in tumor necrosis compared to the untreated cohort (*p* = 0.0038 and *p* = 0.0018, respectively; Fig. 2B). Compared to NHS-IL-12 monotherapy (blue symbols), both docetaxel monotherapy and docetaxel + NHS-IL-12 combination therapy showed a significant increase in necrosis, indicating docetaxel is sufficient to induce tumor necrosis alone (red symbols; *p* = 0.0242, purple symbols; *p* = 0.0094; Fig. 2B). Importantly, this increase in necrosis was not dependent on tumor volume, but dependent on treatment. At end of study, animals treated with docetaxel alone, or in combination with NHS-IL-12, had the highest levels of necrosis, ranging from 20 to 35%, while untreated and NHS-IL-12 monotherapy treated animals averaged < 10% tumor necrosis. Additionally, animals receiving combination therapy had the smallest tumor volumes at date of harvest (Fig. 2C).

The levels of peripheral and intratumoral NHS-IL-12 in the various treatment cohorts were determined via human Fc ELISA. On day 17 post-tumor inoculation, significant accumulation of peripheral NHS-IL-12 was observed in cohorts that received NHS-IL-12 as monotherapy (blue symbols) or in combination with docetaxel (purple symbols) compared to untreated or docetaxel monotherapy (*p* < 0.0001 and *p* < 0.0001, respectively; Fig. 2D). In the tumor microenvironment, increased accumulation of NHS-IL-12 was observed when administered alone (*p* = 0.01) and to a greater extent when it was delivered in combination with docetaxel (purple symbols; *p* = 0.002 when compared to untreated, *p* = 0.001 when compared to docetaxel monotherapy, *p* = 0.12 when compared to NHS-IL-12 monotherapy, Fig. 2E). Overall, these data suggested that tumor necrosis was increased in cohorts receiving docetaxel, and that docetaxel-induced necrosis increased intratumoral retention of NHS-IL-12.

### Antitumor activity of docetaxel and NHS-IL-12 is dependent on tumor targeting

**Fig. 3:**
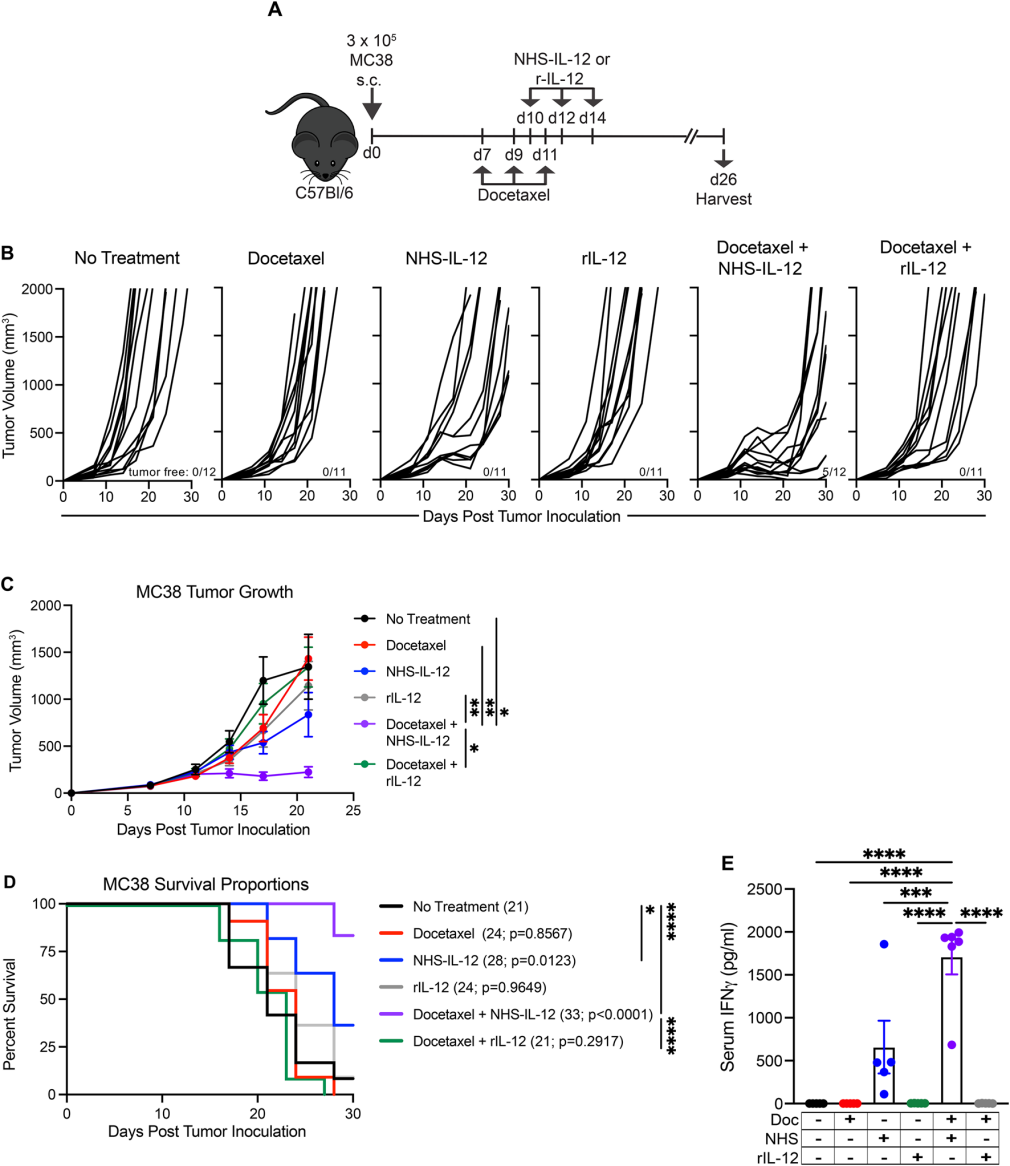
Antitumor activity of docetaxel and NHS-IL-12 is dependent on tumor targeting. (**A**) Graphical representation of experimental design. (**B**) Tumor volumes of MC38 tumor-bearing mice that were untreated or treated with docetaxel, NHS-IL-12, rIL-12, docetaxel + NHS-IL-12 or docetaxel + rIL-12. Numbers at bottom right of graphs indicate animals that were tumor-free at end of study. (**C**) MC38 average tumor volumes of animals treated with docetaxel (red line; *n* = 11), NHS-IL-12 (blue line; *n* = 11), rIL-12 (grey line; *n* = 11), docetaxel + NHS-IL-12 (purple line; *n* = 12), docetaxel + rIL-12 (green line; *n* = 11) and untreated controls (black line; *n* = 12). (**D**) Survival proportions of MC38 tumor-bearing mice treated with docetaxel (red line; *n* = 11), NHS-IL-12 (blue line; *n* = 11), rIL-12 (grey line; *n* = 11), docetaxel + NHS-IL-12 (purple line; *n* = 12), docetaxel + rIL-12 (green line; *n* = 11) and untreated controls (black line; *n* = 12), with median survival and p value relative to untreated control mice indicated in parentheses. (**E**) Serum IFNγ levels in animals treated with docetaxel (red symbols; *n* = 5), NHS-IL-12 (blue symbols; *n* = 5), rIL-12 (grey symbols; *n* = 5), docetaxel + NHS-IL-12 (purple symbols; *n* = 6), docetaxel + rIL-12 (green symbols; *n* = 6) and untreated controls (black symbols; *n* = 5). rIL-12 = recombinant IL-12. Doc = docetaxel. NHS = NHS-IL-12. *p < 0.05, **p < 0.01, ***p < 0.001, ****p < 0.0001

Systemic delivery of IL-12 has resulted in severe dose-limiting toxicities in patients, with tolerated doses providing limited clinical benefit [15, 32]. We first sought to compare the antitumor efficacy of docetaxel in combination with recombinant (r) IL-12 (rIL-12) or in combination with the tumor-targeted NHS-IL-12 in the MC38 tumor model (Fig. 3A). We utilized rIL-12 at an equimolar amount relative to NHS-IL-12. Using the treatment schedule depicted in Fig. 3A, no weight loss was observed with any therapy, either administered as monotherapy or in combination. At end of study, the only treatment group that had tumor-free mice was the docetaxel + NHS-IL-12 combination treated group, with 42% of the animals having undetectable tumors (Fig. 3B). Furthermore, mice that received docetaxel + NHS-IL-12 had significantly smaller tumors in comparison to untreated controls (purple line; *p* = 0.013; Fig. 3C). In contrast, animals that received docetaxel + rIL-12 had no observable differences in mean tumor volume in comparison to untreated control mice (green line; Fig. 3C). The docetaxel + NHS-IL-12 treated group had significantly lower tumor burden in comparison to docetaxel, rIL-12 and docetaxel + rIL-12 treated groups (*p* = 0.0027, *p* = 0.0092, and *p* = 0.0412, respectively; Fig. 3C). Cohorts of mice that received docetaxel (red line), NHS-IL-12 (blue line) or rIL-12 (grey line) monotherapy had no reduction in tumor burden in comparison to untreated controls (Fig. 3C). Additionally, docetaxel + NHS-IL-12 combination therapy significantly extended median survival from 21 days in the untreated cohort to 33 days (purple line; *p* < 0.0001; Fig. 3D). Animals that received docetaxel + rIL-12 had the same median survival as untreated controls (green line; 21 days; *p* = 0.2917; Fig. 3D) and had significantly worse outcome compared to animals that received docetaxel + NHS-IL-12 (*p* < 0.0001; Fig. 3D).

We next measured peripheral IFNγ, a pleiotropic cytokine and a major effector of immunity [33], 12 days after the last dose of all treatments. Cohorts receiving NHS-IL-12 alone or in combination with docetaxel maintained elevated levels of IFNγ (day 26, Fig. 3E). The NHS-IL-12 + docetaxel cohort showed a robust increase in IFNγ in comparison to all other cohorts (purple symbols; *p* < 0.0001; Fig. 3E). These data not only demonstrated the antitumor efficacy of NHS-IL-12 + docetaxel combination over rIL-12, but, moreover, that targeted delivery of IL-12 by NHS76 to the TME contributed to this effect.

### Docetaxel and NHS-IL-12 combination therapy exerts significant antitumor activity in MC38 and EMT6 tumor models

**Fig. 4:**
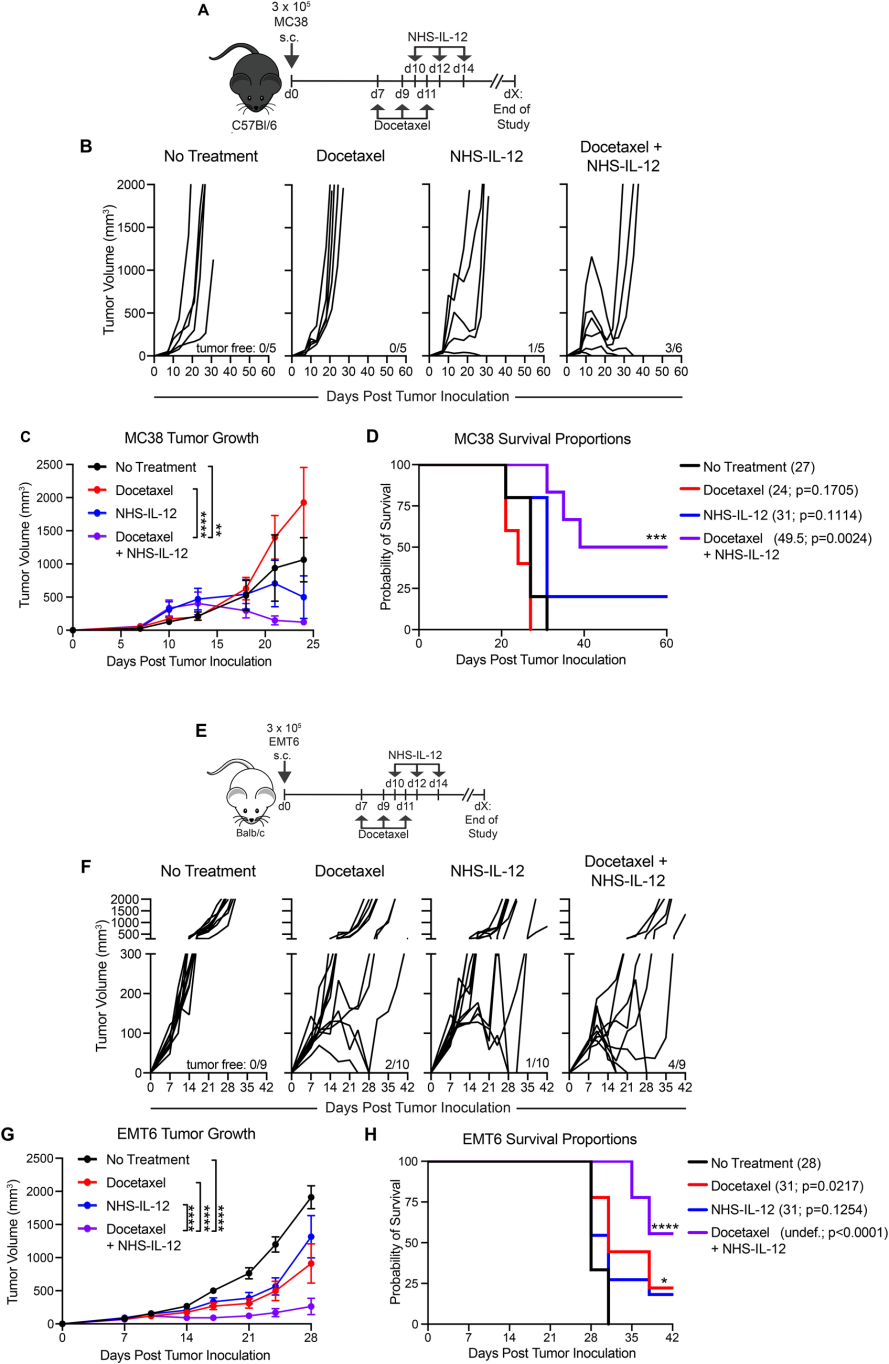
Docetaxel and NHS-IL-12 combination therapy exert significant antitumor activity in the MC38 and EMT6 tumor models. (**A**) Graphical representation of experimental design in the MC38 tumor model. (**B**) Tumor volumes of MC38 tumor-bearing mice in the untreated, docetaxel, NHS-IL-12, and docetaxel + NHS-IL-12 treated cohorts. Numbers at the bottom right of graphs indicate animals that were tumor-free at end of study. (**C**) Average tumor growth curves of MC38 tumor-bearing mice receiving no treatment (black line; *n* = 5), docetaxel (red line; *n* = 5), NHS-IL-12 (blue line; *n* = 5) or docetaxel and NHS-IL-12 combination therapy (purple line; *n* = 6). (**D**) Survival proportions of MC38 tumor-bearing mice receiving no treatment (black line; *n* = 5), docetaxel (red line; *n* = 5), NHS-IL-12 (blue line; *n* = 5) or docetaxel and NHS-IL-12 combination therapy (purple line; *n* = 6) with median survival and p value relative to untreated control mice indicated in parentheses. (**E**) Graphical representation of experimental design using the EMT6 tumor model. (**F**) Tumor volumes of EMT6 tumor-bearing mice in the untreated, docetaxel, NHS-IL-12 and docetaxel + NHS-IL-12 treated cohorts. (**G**) Average tumor volumes of EMT6 tumor-bearing mice receiving no treatment (black line; *n* = 9), docetaxel (red line; *n* = 10), NHS-IL-12 (blue line; *n* = 10) or docetaxel + NHS-IL-12 combination therapy (purple line; *n* = 9). (**H**) Survival proportions of EMT6 tumor-bearing mice with median survival and p value relative to untreated control mice indicated in parentheses. Undef. = undefined. *p < 0.05, **p < 0.01, ***p < 0.001, ****p < 0.0001

Previous studies have shown antitumor activity of NHS-IL-12 in “warm” and “cold” tumors models [12, 31]. Therefore, we next aimed to determine the in vivo efficacy of docetaxel and NHS-IL-12 in combination in the “warm” MC38 colorectal tumor model and “cold” EMT6 breast cancer model. MC38 tumor-bearing mice were randomized, and treatment was initiated on day 7. In this model, we observed 20% of animals treated with NHS-IL-12 monotherapy were tumor-free, whereas 50% of animals that received docetaxel + NHS-IL-12 combination therapy were tumor-free (Fig. 4B). When tumor burden in individual cohorts was evaluated, animals treated with docetaxel + NHS-IL-12 had significantly reduced tumor volumes in comparison to docetaxel monotherapy and untreated cohorts (*p* < 0.0001, *p* = 0.031, respectively; Fig. 4C). In this model, we observed that animals left untreated succumbed to tumor burden approximately 27 days post tumor inoculation (black line; Fig. 4D). Animals that received either docetaxel or NHS-IL-12 as monotherapies did not exhibit a significant survival advantage, with each group reaching a median overall survival of 24 (red line; *p* = 0.1705) and 31 days (blue line; *p* = 0.1114), respectively (Fig. 4D). In contrast, when docetaxel and NHS-IL-12 were administered in combination, median survival was significantly extended to 49.5 days (purple line; *p* = 0.0024; Fig. 4D).

Next, the antitumor activity of docetaxel + NHS-IL-12 combination therapy in the EMT6 breast cancer model was assessed (graphical representation of experimental design in Fig. 4E). Docetaxel and NHS-IL-12 monotherapy cohorts had 20% and 10% of animals that were tumor-free at end of study, respectively (Fig. 4F). However, the greatest frequency of tumor-free animals at end of study were those treated with docetaxel and NHS-IL-12 in combination (44%; Fig. 4F). Furthermore, when we evaluated tumor burden in the EMT6 model, cohorts that received docetaxel + NHS-IL-12 had significantly lower tumor volumes compared to untreated group (purple line; *p* < 0.0001; Fig. 4G). The combination therapy also exerted a significant decrease in tumor burden compared to docetaxel (red line; *p* < 0.001) and NHS-IL-12 (blue line; *p* < 0.001) monotherapies. Contrary to what we observed in the MC38 model, docetaxel monotherapy significantly extended median survival from 28 days in the untreated cohort to 31 days (red line; *p* = 0.0217; Fig. 4H). While median survival in the NHS-IL-12 monotherapy cohort was also extended to 31 days, there was no significant advantage in comparison to untreated controls (blue line; *p* = 0.1254; Fig. 4H). Consistent with our observations in the MC38 model, cohorts that received docetaxel + NHS-IL-12 in combination had an undefined median survival that was significantly extended in comparison to untreated control mice (purple line; *p* < 0.0001; Fig. 4H). These data, taken together, demonstrate that docetaxel + NHS-IL-12 combination therapy achieved a significant antitumor effect on a “warm” colon and a “cold” breast cancer model.

### Docetaxel and NHS-IL-12 combination therapy exerts significant antitumor activity independent of PD-L1 tumor expression

**Fig. 5:**
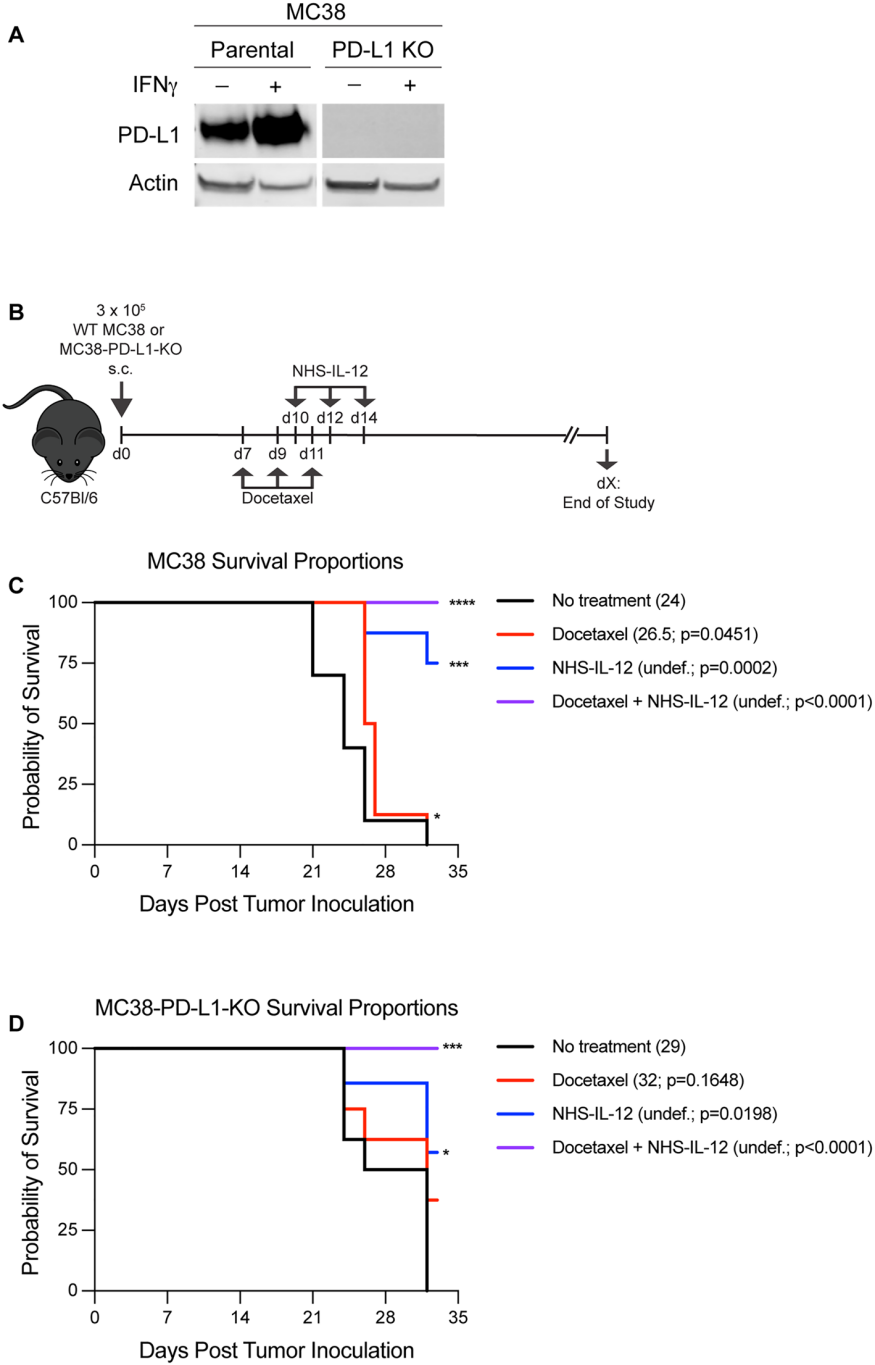
Docetaxel and NHS-IL-12 combination therapy exerts significant antitumor activity independent of PD-L1 tumor expression. (**A**) Western blot analysis of PD-L1 expression of wild type parental MC38 and MC38-PD-L1 KO cells with and without IFNγ treatment. (**B**) Graphical representation of experimental design using the MC38 and MC38-PD-L1 KO tumor models. Survival proportions of (**C**) MC38 tumor-bearing mice and (**D**) MC38-PD-L1 KO mice treated with docetaxel (red line), NHS-IL-12 (blue line), docetaxel + NHS-IL-12 (purple line), and untreated controls (black line). Median survival and p values relative to untreated control mice indicated in parentheses. PD-L1 = programmed death ligand 1; KO = knockout; Undef. = undefined. *p < 0.05, **p < 0.01, ***p < 0.001, ****p < 0.0001

Primary resistance to PD-1/PD-L1 checkpoint blockade is one of the many challenges faced by the immunotherapy field [34]. We sought to interrogate the NHS-IL-12 plus docetaxel combination therapeutic efficacy using a MC38-PD-L1 KO model. CRISPR/Cas9 technology was utilized to generate PD-L1-deficient MC38 cells and the loss of PD-L1 was confirmed by the inability of the cells to express PD-L1 even after strong in vitro IFN-γ stimulation (Fig. 5A).

MC38 and MC38-PD-L1 KO tumor-bearing mice were treated as described in Fig. 5B. Consistent with our previous results (Figs. 3D and 4D), MC38 tumor-bearing mice treated with docetaxel + NHS-IL-12 in combination had improved overall survival compared to the untreated group (purple line; *p* < 0.0001; Fig. 5C). At end of the study, the estimated median survival was not reached for the docetaxel + NHS-IL-12 cohort, while the median survival for the untreated cohort was 24 days. Similarly, 100% of the MC38-PD-L1 KO tumor-bearing mice that received docetaxel + NHS-IL-12 were alive at the end of the study resulting in a significantly increased survival benefit when compared to the untreated cohort (29 days) (purple line; *p* = 0.0001; Fig. 5D). These data indicated that docetaxel + NHS-IL-12 combination therapy significantly extended survival in mice with MC38 tumors, regardless of PD-L1 expression.

### Docetaxel and NHS-IL-12 combination therapy requires CD8^+^***T cells for efficient antitumor activity***

**Fig. 6:**
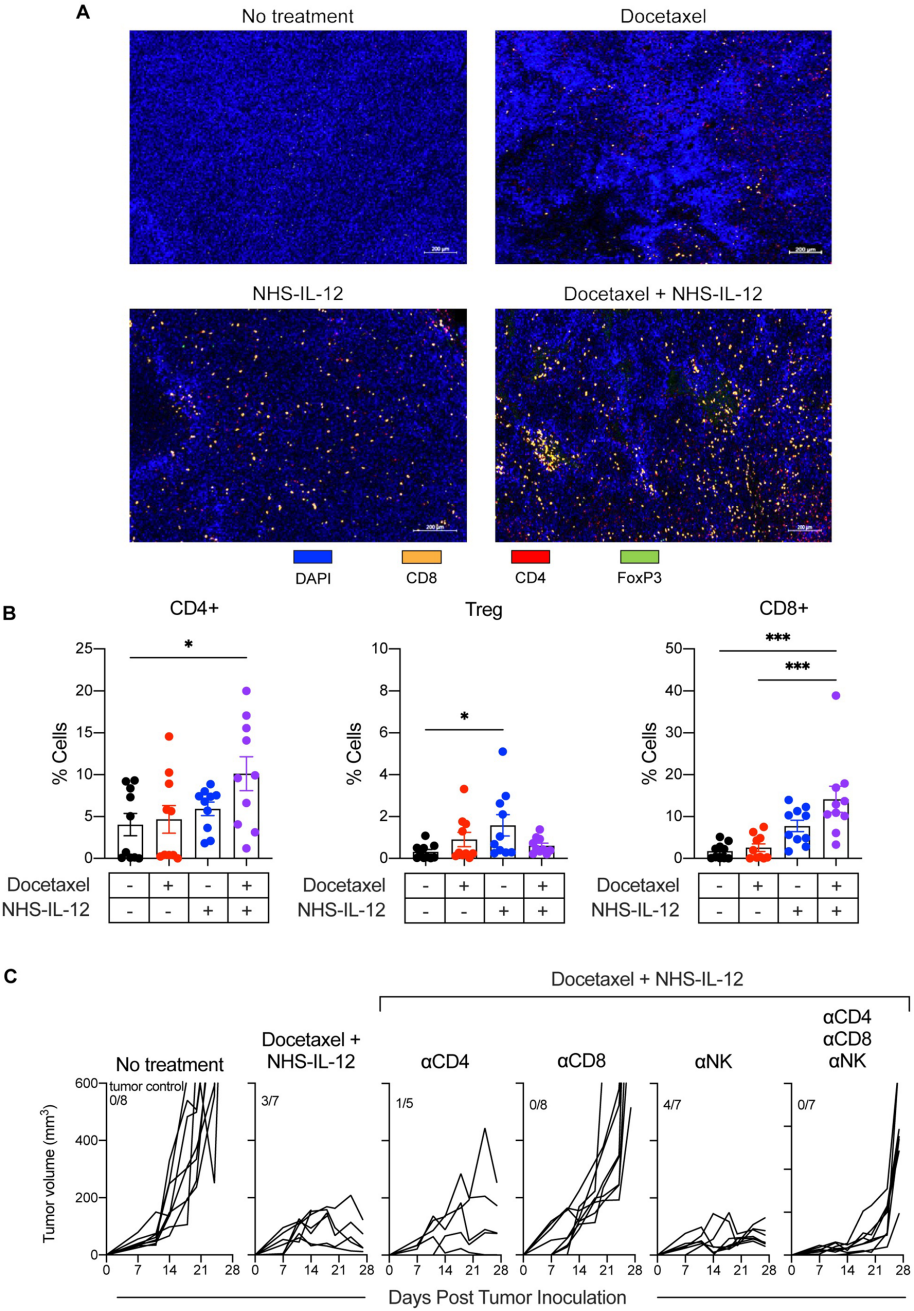
Docetaxel and NHS-IL-12 combination therapy requires CD8^+^ T cells for efficient antitumor activity. (**A**) Representative immunofluorescence staining of MC38 tumors from untreated animals and animals treated with docetaxel, NHS-IL-12, and docetaxel + NHS-IL-12, 22 days post tumor implantation. Staining includes DAPI (blue), CD8^+^ T cells (orange), CD4^+^ T cells (red), and FoxP3^+^ regulatory T cells (green). (**B**) Quantification of CD4^+^ T cells, Tregs (CD4^+^FoxP3^+^) and CD8^+^ T cells. (**C**) Tumor volumes of MC38 tumor-bearing mice in the untreated and docetaxel + NHS-IL-12 treated cohorts that were depleted of CD4^+^ T cells, CD8^+^ T cells, and/or NK cells. Numbers at the top left of graphs indicate animals capable of controlling tumor growth (tumors < 50mm^3^) at end of study. NK = natural killer. *p < 0.05, **p < 0.01, ***p < 0.001, ****p < 0.0001

To assess tumor-infiltrating lymphocytes (TILs) in the TME, MC38 tumor-bearing mice were treated as described in Fig. 4A. On day 22 post tumor implantation, tumors were fixed, paraffin-embedded, and stained using multiplex immunofluorescence. Our results showed that tumors from the docetaxel and NHS-IL-12 monotherapy groups had similar frequencies of CD4^+^ T cells as the untreated group (Fig. 6A, B, red and blue symbols, respectively). In contrast, tumors from the docetaxel + NHS-IL-12 combination therapy cohort showed a significant increase in CD4^+^ T cell frequency as compared to the untreated cohort (Fig. 6A, B, purple symbols; *p* = 0.0358). Analysis of CD4 and FoxP3 staining showed that neither docetaxel monotherapy nor docetaxel + NHS-IL-12 combination therapy altered Treg frequency in the tumor (Fig. 6A, B). Treatment with the docetaxel + NHS-IL-12 combination therapy cohort yielded a statistical increase in the frequency of CD8^+^ T cells compared to the untreated cohort (Fig. 6B, purple symbols; *p* = 0.0001), and animals treated with docetaxel monotherapy (Fig. 6B, purple symbols, *p* = 0.0003).

Docetaxel + NHS-IL-12 combination therapy failed to provide tumor control following CD4^+^ T cell depletion (*p* = 0.9968; Fig. 6C), with few animals capable of controlling tumor growth (number of animals with less than 50mm^3^ reduced from 3/7 to 1/5). As the immunofluorescence data (Fig. 6B) suggested that the therapy mediated by docetaxel and NHS-IL-12 was associated with increased CD8^+^ cell frequency, we next interrogated the functional contribution of T cell subsets by in vivo depletion studies. Upon depletion of CD8^+^ T cells, docetaxel + NHS-IL-12 combination therapy failed to control tumor growth (*p* < 0.0001; Fig. 6C). In contrast, removal of NK cells did not alter the efficacy of docetaxel + NHS-IL-12 combination therapy (*p* = 0.9900; Fig. 6C). Finally, as expected, depletion of CD4^+^ T cells, CD8^+^ T cells and NK cells in mice receiving docetaxel + NHS-IL-12 combination therapy abrogated all antitumor efficacy previously observed with the treatment (*p* < 0.0001; Fig. 6C). Taken together, these findings confirmed the requirement for CD8^+^ T cells in the TME to exert the antitumor effects delivered by docetaxel + NHS-IL-12 combination therapy in the “warm” MC38 tumor model.

### Docetaxel and NHS-IL-12 combination therapy creates an immunostimulatory tumor microenvironment

**Fig. 7:**
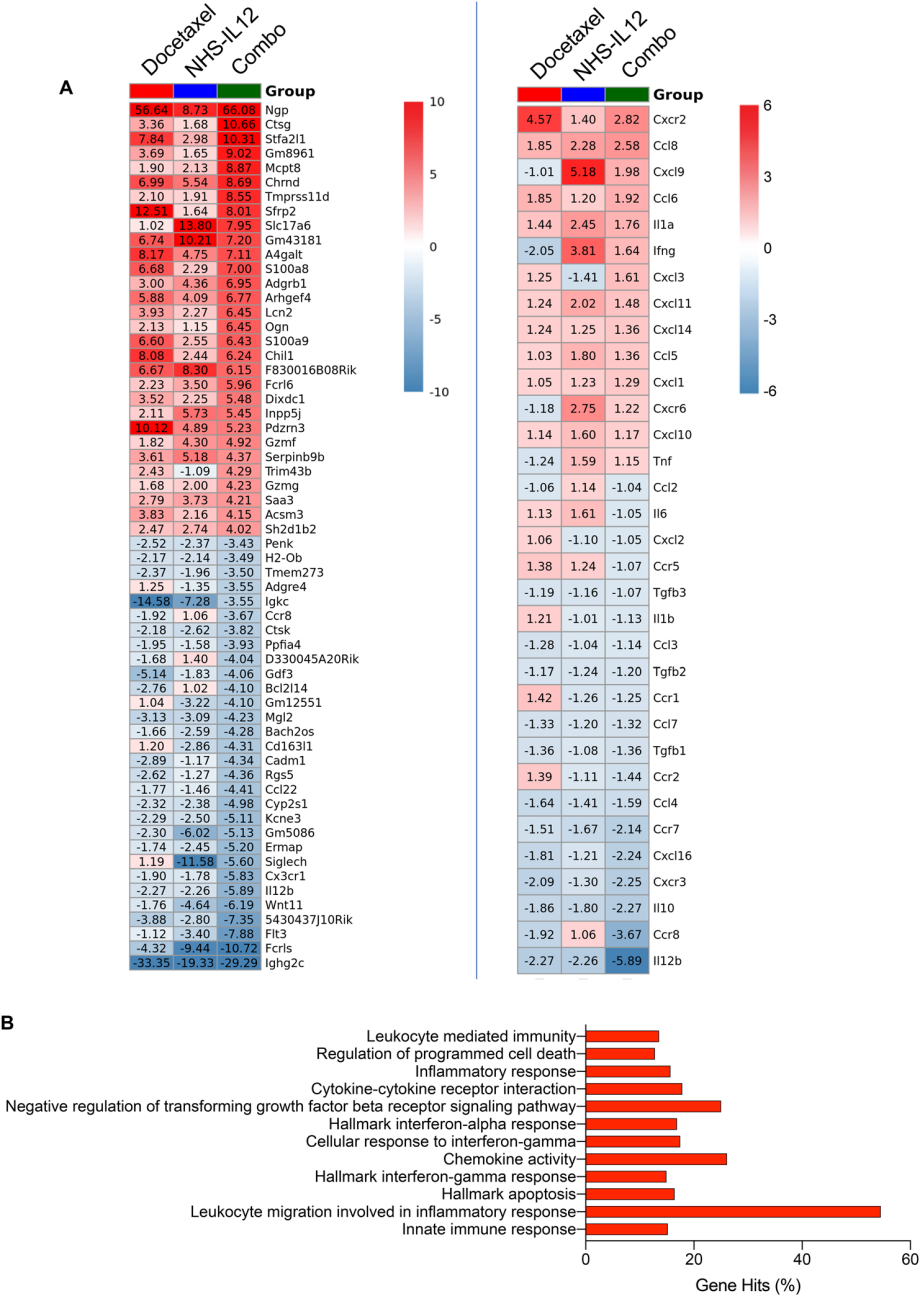
Docetaxel and NHS-IL-12 combination therapy sets up an immune-stimulatory tumor microenvironment. MC38 tumor-bearing mice treated as in Fig. 3A were sacrificed on day 17 post-tumor implant and tumor whole transcriptome analysis was performed. Gene set enrichment analysis was carried out for docetaxel and NHS-IL-12 combination therapy versus untreated tumors. (**A**) Heatmap of bulk-RNA-seq data collected on day 17 post-treatment showing the top 30 upregulated and downregulated transcripts in MC38 tumors treated with monotherapy versus combination therapy, normalized to tumor expression with PBS treatment. (**B**) Transcriptomic analysis of combination-treated tumors shows significant enrichment in pathways associated with tumor suppression. Data represent one experiment, *n* = 3–4 mice/group. Fisher’s test for pathways applied top 10% genes with p value cut-off of *p* = 0.05. GO, gene ontology; KEGG, Kyoto Encyclopedia of Genes and Genomes

To better understand the mechanisms mediating tumor clearance, we performed RNA analysis of bulk tumors on each treatment modality (docetaxel, NHS-IL-12, and docetaxel + NHS-IL-12). Gene expression of *F830016B08Rik* (IFNγ-inducible GTPase), *Gzmf* (granzyme f), and *Gzmg* (granzyme g) was upregulated at the transcript level with combination therapy (Fig. 7A). These observations are consistent with findings in Fig. 3E, which showed a significant increase in peripheral IFNγ levels in animals treated with docetaxel + NHS-IL-12 combination therapy compared to NHS-IL-12 monotherapy. Assessment of peripheral cytokines in both MC38 (Supplementary Fig. 1A) and EMT6 (Supplementary Fig. 1B) tumor models revealed that the combination treatment with docetaxel + NHS-IL-12 yielded increased levels of the proinflammatory cytokines IFNγ, IL-12, and tumor necrosis factor α (TNFα). Monotherapy administration of NHS-IL-12 in the MC38 model resulted in increased peripheral KC/GRO (Supplementary Fig. 1A, blue symbols), whereas increased KC/GRO in the EMT6 model only occurred following combination treatment with docetaxel + NHS-IL-12 (Supplementary Fig. 1B, purple symbols).

Many mediators of innate immunity were upregulated at the transcript level with combination therapy (e.g., *Ctsg*, *S100a8*, *S100a9*; Fig. 7A), contributing to positive regulation of the immune response. This positive regulation of the immune response might lead to an increase in phagocytosis of necrotic debris generated by docetaxel (e.g., *Ctsg*, *Mcpt8*, *S100a8*, *S100a9*, *Lcn2*, *Chil1*, *Saa3*; Fig. 7A) [35].

We also performed Kyoto Encyclopedia of Genes and Genomes (KEGG) and gene ontology (GO) enrichment analysis to identify relevant enriched pathways and biological functions that were activated in docetaxel + NHS-IL-12 combination therapy relative to untreated tumors. KEGG/GO pathway analysis revealed the leukocyte migration in inflammatory response, innate immune response, inflammatory response, chemokine activity, and leukocyte mediated immunity related pathways were more activated in tumors treated with combination therapy (Fig. 7B). Consistent with observed increases in peripheral IFNγ levels in docetaxel + NHS-IL-12 treated cohorts (Fig. 3E), we also detected activation of the hallmark IFNγ response, hallmark IFNα response, and cellular response to IFNγ related pathways (Fig. 7B). GO analysis also showed significant activation of the negative regulation of transforming growth factor beta receptor (TGFβR) signaling pathway (Fig. 7B). In addition, docetaxel + NHS-IL-12 combination therapy activated regulation of programmed cell death and hallmark apoptosis pathways (Fig. 7B).

## Discussion

Necrosis is a common pathologic feature of solid tumors that has often been utilized as a negative prognostic indicator. Tumor necrosis may be indicative of an aggressive malignant phenotype where the tumor outgrows its vascular supply, resulting in hypoxia, accumulation of metabolic waste, and eventually necrosis [3, 4, 36]. Indeed, a retrospective study in small hepatocellular carcinoma (sHCC) correlated tumor necrosis with poorer cancer-specific overall survival and recurrence-free survival (RFS) [28]. Another study found the presence of tumor necrosis in papillary renal cell carcinoma (RCC) was an independent negative prognostic factor for metastasis-free survival [17]. While the utility of tumor necrosis as a parameter to predict cancer aggressiveness remains inconclusive, clinical benefit of chemotherapy- or radiation-induced tumor necrosis is well documented [37, 38]. Importantly, the occurrence of treatment-induced tumor necrosis (i.e., neoadjuvant chemotherapy) has been considered a positive correlative, with several studies reporting high levels of tumor necrosis following chemotherapy treatment resulted in significantly increased disease-free survival (DFS) [39–41].

Our results demonstrated docetaxel is capable of inducing necrosis in the murine MC38 colon cancer model (Figs. 1B, 2B) in a dose-dependent manner (Fig. 1D). Additionally, our data revealed docetaxel-induced necrosis is not dependent on tumor volume, as we reported the smallest tumors in our docetaxel + NHS-IL-12 combination treatment group had the highest level of necrosis (Fig. 2C).

Tumor-targeting cytokines, like NHS-IL-12, have the potential to diminish the toxicities observed with rIL-12 [12]. Indeed, NHS-IL-12 administration in a first-in-human Phase I clinical trial in patients with advanced solid malignancies revealed no major safety concerns [23]. This trial determined the maximum tolerated dose (MTD) (16.8 µg/kg) and reported an increase in IFNγ, with an associated increase in IL-10 following NHS-IL-12 administration. This study did not report objective tumor responses, but five subjects had durable stable disease (range: 6–30+ months). Upon publication of these results, NHS-IL-12 was recommended for a Phase II clinical trial [23].

Our data confirmed that docetaxel induced necrosis at a standard-of care dose, which allowed for a significant increase in intratumoral NHS-IL-12 in the combination treatment group (Fig. 2D and E). These data support and extend the observations of Hicks et al. [20], which showed that NHS-IL-12 was retained in necrotic regions of tumors induced by entinostat, a class I HDAC inhibitor [20].

It has been described previously that docetaxel in combination with high-dose NHS-IL-12 improved antitumor activity in the MC38 murine model of colorectal cancer [18]. We presented data herein that low dose NHS-IL-12 in combination with docetaxel is sufficient to induce robust and durable antitumor activity. To interrogate the robustness of the NHS-IL-12 plus docetaxel therapy, we deliberately chose the “cold” tumor model EMT6 with the goal to induce T cell infiltration to “cold” tumors. In the MC38 tumor model, the therapeutic effect of the combination treatment was initially observed on day 14 post-tumor inoculation (Fig. 4B). This contrasts with that of EMT6, where the therapeutic effect of the combination treatment was initially observed on day 7 post-tumor inoculation (Fig. 4F). We believe that the similarity in overall antitumor activity in both tumor models in terms of the antitumor efficacy could be attributed to the possibility that the NHS-IL-12 regimen may modulate the immune compartment of a “cold” tumor into a “warm” one. A previous study by our team showed that NHS-IL-12 monotherapy has the potential to increase TILs in the TME, such as NK cells and CD8^+^ T cells [18]. Those data are in agreement with our data where NHS-IL-12 increases CD8^+^ T cells (Fig. 6A).

In addition to controlling tumor growth, we also reported a 50% and 44.5% cure rate in the MC38 and EMT6 models, respectively (Fig. 4B, F). The advantage of combination therapy with docetaxel + NHS-IL-12 was further emphasized by the significant increase in overall survival in MC38, EMT6 and MC38-PD-L1 KO tumor models (Figs. 4B, H, 5C, D). Importantly, our combination therapy significantly increased survival in our checkpoint resistant model (MC38-PD-L1 KO; Fig. 5D), thereby providing rationale to use this strategy in patients who are unresponsive to anti-PD-1/PD-L1 therapy.

Combination therapies using several IO agents have the ability to engage, expand, enable, and evolve the immune response in “warm” and “cold” tumor models [31]. Our study confirmed docetaxel + NHS-IL-12 combination therapy significantly increased CD4^+^ and CD8^+^ T cell populations in the TME (Fig. 6A, B). Furthermore, detailed analysis of the transcriptomic profile showed upregulation of selected genes in the following activated pathways: leukocyte migration in inflammatory response (*s100a8*), innate immune response (*Lcn2*, *Ccl8*, *Gzmf*, *Gzmg*, *Sh21b2*), inflammatory response (*Chil1*, *Cxcr1*, *Cxcr2*, *Tnfrsf9*), chemokine activity (*Ccl8*), and leukocyte-mediated immunity (*Chil1*, *Ctsg*, *Gzmf*, *Cxcr1*, *Cxcr2*, *Lcn2*, *Serpinbnb*) (Fig. 7A, B). This, accompanied with the increase in CD8^+^ T cell-attracting chemokine genes (*Cxcl9*, *Cxcl10*, *Ccl5*; Fig. 7A), correlated with increased infiltration of CD8^+^ T cells in tumors that received docetaxel + NHS-IL-12 combination therapy (Fig. 6A, B).

Docetaxel + NHS-IL-12 combination therapy is being investigated in a Phase I/II clinical trial for patients with metastatic castration sensitive/resistant prostate cancer (NCT04633252) [24]. While no results have been published at the time of this writing, this study is expected to report tumor necrosis and immune infiltrates from collected biopsies as a measure to predict response/failure to therapy. The data presented herein provide strong rationale to study docetaxel + NHS-IL-12 combination therapy in patients who are resistant to checkpoint blockade therapy.

### Supplementary Information

Below is the link to the electronic supplementary material.

Supplementary file1 (PDF 224 kb)

## Data Availability

Data will be made available upon reasonable request**.**
