# Supplementary material for: Exploiting docetaxel-induced tumor cell necrosis with tumor targeted delivery of IL-12
Source: Cancer Immunol Immunother. 2023 May 11;72(8):2783–97. doi: 10.1007/s00262-023-03459-7 (PMC10361896; doi:10.1007/s00262-023-03459-7)
Supplement: Supplementary file 1 — Supplementary file1 (PDF 224 kb) [file 262_2023_3459_MOESM1_ESM.pdf]

Supplemental Figure 1

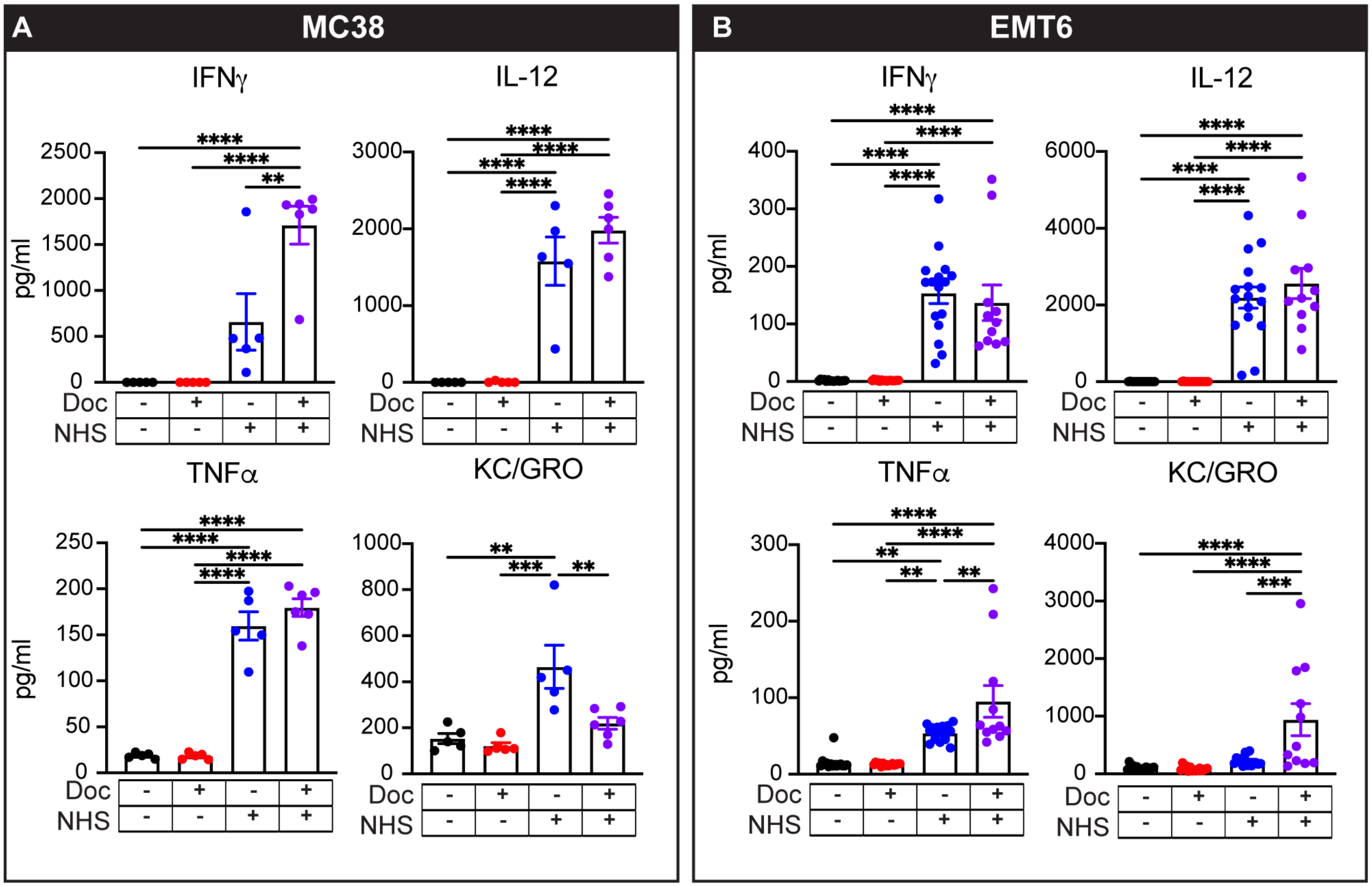

Supplemental figure 1. Docetaxel and NHS-IL-12 combination therapy increase pro-inflammatory cytokines. Quantification of peripheral IFN-γ, IL-12, TNFα, and KC/GRO in the **(A)** MC38 tumor model in animals treated with docetaxel (red symbols; *n*=5), NHS-IL-12 (blue symbols; *n*=5), docetaxel + NHS-IL-12 (purple symbols; *n*=6) and untreated controls (black symbols; *n*=5) on day 16 post tumor inoculation. Quantification of peripheral IFNγ, IL-12, TNFα, and KC/GRO in the **(B)** EMT6 tumor model in animals treated with docetaxel (red symbols; *n*=15), NHS-IL-12 (blue symbols; *n*=15), docetaxel + NHS-IL-12 (purple symbols; *n*=11) and untreated controls. Doc = docetaxel. NHS = NHS-IL-12. *= p<0.05, **= p<0.01, ***=p<0.001, ****=p<0.0001.
